# Peptide-Based Strategies Against *Mycobacterium tuberculosis* Covering Immunomodulation, Vaccines, Synergistic Therapy, and Nanodelivery

**DOI:** 10.3390/ph18101440

**Published:** 2025-09-25

**Authors:** Cesar Augusto Roque-Borda, Subham Kumar Vishwakarma, Oswaldo Julio Ramirez Delgado, Heitor Leocadio de Souza Rodrigues, Laura M. D. Primo, Isabella Cardeal Campos, Tulio Spina de Lima, João Perdigão, Fernando Rogério Pavan

**Affiliations:** 1 School of Pharmaceutical Sciences, São Paulo State University (UNESP), Araraquara 14800-903, São Paulo, Brazil; 2 iMed.ULisboa–Institute for Medicines Research, Faculty of Pharmacy, University of Lisbon, 1649004 Lisbon, Portugal; 3 School of Agricultural and Veterinary Sciences, São Paulo State University (UNESP), Jaboticabal 14884-900, São Paulo, Brazil

**Keywords:** antimicrobial peptides, *Mycobacterium tuberculosis*, peptide-based therapy, drug design, nanodrug delivery systems

## Abstract

Tuberculosis (TB), caused by *Mycobacterium tuberculosis* (MTB), remains one of the most devastating infectious diseases worldwide, with rising multidrug resistance limiting the effectiveness of conventional treatments. Novel therapeutic approaches are urgently needed to complement or replace existing regimens. Among emerging candidates, antimicrobial peptides (AMPs) stand out as versatile molecules capable of exerting direct antimycobacterial effects while also modulating the host immune response. This review explores peptide-based strategies against TB, with a focus on four major axes of innovation. First, we examine host-directed pathways, including the vitamin D–cathelicidin axis and other immunomodulatory mechanisms and their regulatory role in the induction of endogenous AMPs such as cathelicidin LL-37, which contributes to host-directed defense. Second, we discuss peptide-based vaccines designed to elicit robust and durable protective immunity, representing a complementary alternative to classical vaccine approaches. Third, we highlight the synergistic potential of AMPs in combination with first-line and second-line anti-TB drugs, aiming to restore or enhance bactericidal activity against resistant strains. Finally, we analyze technological platforms, including nanocarriers and inhalable formulations, that enable targeted pulmonary delivery, improve peptide stability, and enhance bioavailability. By integrating molecular design, immune modulation, and advanced delivery systems, peptide-based strategies provide a multifaceted approach to overcoming the limitations of current TB therapy. Collectively, these advances position AMPs not only as promising standalone agents but also as key components in combination and host-directed therapies, with strong potential to reshape the future clinical management of tuberculosis.

## 1. Introduction

Tuberculosis (TB) remains one of the most severe infectious diseases globally, accounting for millions of new cases and deaths every year [1]. According to the World Health Organization (WHO), in 2023, around 10.8 million people worldwide contracted tuberculosis, including 6.0 million men, 3.6 million women, and 1.3 million children. In 2024, TB continued to be one of the deadliest infectious diseases, with an estimated 10.6 million new cases. The disease claimed 1.3 million lives, including 167,000 deaths among people living with HIV [1]. Despite decades of chemotherapeutic use, *Mycobacterium tuberculosis* (MTB) continues to pose a major threat to public health, particularly in low- and middle-income countries where treatment adherence is often difficult to sustain [2]. The complexity of TB pathology, its long treatment regimens, and the socioeconomic impact of disease burden underscore the urgent need for innovative therapeutic strategies that can complement or replace conventional drugs [3].

The cornerstone of TB therapy has long relied on the combined administration of first-line antibiotics such as isoniazid, rifampicin, pyrazinamide, and ethambutol. However, the increasing prevalence of multidrug-resistant (MDR) and extensively drug-resistant (XDR) strains has dramatically reduced the effectiveness of these regimens [4]. Even recently approved agents, including bedaquiline and delamanid, are already facing reports of emerging resistance. These developments highlight a critical therapeutic gap: the current arsenal is insufficient to keep pace with the adaptive capacity of MTB [5,6].

Within this context, antimicrobial peptides (AMPs) have emerged as a compelling alternative. These molecules are produced by a wide range of organisms as part of their innate defense mechanisms and exhibit potent activity against diverse pathogens, including MTB [7]. Unlike conventional antibiotics, which often target single molecular pathways, AMPs display multifaceted modes of action, including the disruption of mycobacterial membranes, interference with metabolic processes, and modulation of host immunity. This multifaceted behavior reduces the probability of resistance development and positions AMPs as versatile candidates in anti-TB therapy [8].

One of the most intriguing dimensions of peptide biology in TB is their integration into host-directed therapy (HDT). Endogenous peptides, such as cathelicidin LL-37, are tightly regulated by the vitamin D pathway, linking metabolic and immune responses to antimicrobial activity [9,10]. This connection has stimulated research into the use of vitamin D supplementation and synthetic AMP analogs as immunomodulatory interventions. By reinforcing autophagy, enhancing macrophage activity, and promoting intracellular killing of MTB, AMPs offer a strategy that not only targets the pathogen but also strengthens host resilience [11,12].

In parallel, peptides are being investigated as vaccine components. Their ability to provide targeted immune stimulation, combined with advances in epitope mapping and computational design, has generated promising candidates capable of eliciting T-cell–mediated protection [13]. Unlike traditional vaccines, peptide-based formulations can be engineered with high precision, reducing the risk of adverse reactions while maintaining immunogenic potency. This precision design aligns with the urgent need for vaccines capable of addressing diverse MTB lineages and resistant strains.

Finally, the translational success of peptide therapeutics depends largely on overcoming pharmacokinetic barriers. AMPs are susceptible to enzymatic degradation and may display limited systemic stability. To address these challenges, significant efforts have been directed toward nanocarrier systems and inhalable formulations that allow targeted pulmonary delivery. Such platforms not only protect peptide integrity but also enhance local concentration at the site of infection. Moreover, combinations of AMPs with conventional drugs have demonstrated synergistic effects, improving bactericidal outcomes while potentially mitigating resistance. Taken together, these advances indicate that peptide-based strategies, encompassing host-directed mechanisms, synergistic therapies, and advanced delivery systems, represent a promising frontier in the fight against tuberculosis.

## 2. Host-Directed Antimicrobial Peptides in Tuberculosis: A Promising Therapeutic Avenue

Host-directed antimicrobial peptides (HDPs) represent an alternative strategy to control MTB by modulating the host immune response, and in some cases, acting synergistically with anti-TB drugs [8]. This approach has gained attention because the development of new TB drugs is costly and time-consuming [14]. Endogenous peptides, such as defensins and the cathelicidin LL-37, play key roles in the host defense against MTB [15,16]. HDPs expressed by host innate immune cells display diverse immunomodulatory functions, including cell lysis, regulation of inflammatory responses, enhancement of host defense, and the formation of autophagosomes and phagolysosomes [8]. In addition, synthetic analogues have been developed to modulate immunity [17].

At the infection site, neutrophils arrive and release human neutrophil α-defensins (HNPs), promoting mycobacterial activity, especially HNPs 1–3 [18]. Additionally, HNPs can activate macrophages, which subsequently secrete TNF-α and IFN-γ, thereby improving phagocytosis [19]. In contrast, β-defensins are mainly expressed by epithelial cells [20]. The human β-defensin 1 (hBD1) has been shown to enhance mycobactericidal activity when combined with isoniazid, which has significantly reduced MTB [21]. Furthermore, hBD-2 expression occurs in response to the induction by pro-inflammatory cytokines [22] and has demonstrated mycobactericidal activity in infected macrophages [23]. Moreover, hBD4 induction can be observed in the presence of both IL-1β and vitamin D, influencing MTB intramacrophage survival [24].

During infection, Toll-like receptors (TLRs) participate in both innate and adaptive immune responses, promoting the initiation of the latter [25]. TLR stimulation, particularly by TLR-2, TLR-4, and TLR-9, leads to the significant production of the human cathelicidin LL-37 by neutrophils, epithelial cells, and alveolar macrophages [25,26]. In macrophages, LL-37 contributes to maintaining the balance between apoptosis and cell necrosis [27] and has demonstrated immunomodulatory properties. This is characterized by an increase in IL-10 and TGF-β and a decrease in TNF-α and IL-17, thereby controlling the inflammatory process and, consequently, limiting tissue damage and disease progression [28].

Regarding synthetic analogues, innate defense regulators display immunomodulatory characteristics in the host, including the expression of antimicrobial agents, the release of chemokines, and the reduction in inflammation [8,17]. The IDR peptides HH2 and 1018 have been associated with the induction of macrophage chemokines MCP-1 and Gro-α and have demonstrated a reduction in bacterial load in the lungs of infected mice with the drug-sensitive MTB strain H37Rv (ATCC no.25618) and an MDR strain [17]. Also, the Hcl2 peptide, derived from the human mitochondrial cytochrome c oxidase subunit 3, has already demonstrated the capacity to bind to the MTB virulence factor ESAT-6, leading to the disruption of the ESAT-6/CFP10 complex and, consequently, reducing MTB survival [29].

Therefore, HDPs are a promising option for TB treatment. However, more studies are needed regarding pharmacokinetics, including delivery mechanisms, stability, and half-life. Natural antimicrobial peptides are susceptible to physiological salt concentrations and protease degradation, leading to a short serum half-life. Additionally, there is a lack of selectivity and a potential toxicity and immunogenicity when produced in other organisms [30]. Furthermore, effectiveness, safety, and optimal implementation of HDPS should be assessed with clinical trials, which are still scarce [31]. Additionally, the development of new anti-TB drugs should be evaluated under rigorous guidelines; the European Medicines Agency (EMA) requires the establishment of the minimal inhibitory concentration according to the EUCAST reference method, as well as pharmacokinetics, pharmacodynamics, and clinical outcomes [32].

Beyond host-directed AMPs, it is important to recognize that HDPs represent a subset within the broader class of antimicrobial peptides, many of which display overlapping yet distinct mechanisms of action. Several AMPs of non-host origin, including bacterially and synthetically derived sequences, also exert immunomodulatory properties by shaping cytokine release, promoting antigen presentation, or enhancing T cell responses. This diversity highlights the potential of integrating peptide-based strategies not only as therapeutic agents but also as immunogens. Indeed, peptide epitopes derived from MTB proteins, or synthetic constructs optimized for major histocompatibility complex (MHC) binding, have been evaluated as vaccine candidates to elicit protective adaptive responses. Thus, while this review emphasizes HDPs as dual-acting molecules that bridge innate immunity and antimicrobial activity, it is essential to situate them within the wider landscape of peptide-based interventions, encompassing both immunotherapeutic approaches and next-generation vaccine design.

## 3. Synergy Between Conventional TB Drugs and Antimicrobials Peptides

The global rise of MDR and XDR MTB strains has created an urgent need for innovative therapeutic approaches that can overcome resistance and enhance treatment efficacy. Since monotherapy has been shown to favor the selection of drug-resistant mycobacteria, combination therapy has been established as the gold standard in tuberculosis management [33]. Within this context, the use of synergistic drug combinations, particularly those involving AMPs and conventional anti-TB agents, has gained attention as a promising strategy in preclinical studies, as summarized in Table 1 [34,35,36].

Several AMPs have demonstrated MIC values comparable to those of first-line anti-TB drugs in vitro [33,37,38]. When used in combination, these peptides often display synergistic interactions with antibiotics, leading to marked reductions in the MICs of both agents. This not only enhances antimicrobial efficacy but also enables dose reduction, which may lower systemic toxicity and delay the onset of resistance [35]. For example, a small fraction (1/16 of its MIC value) of the peptide M(LLKK2)M was reduced by half the concentration of rifampicin required to inhibit rifampicin-resistant MTB. Moreover, this synergistic combination also delayed the development of additional resistance to rifampicin [38]. Similarly, the combination of peptide D-LAK with isoniazid was able to overcome resistance to this antibiotic in three MDR MTB strains [34]. Considering that resistance to rifampicin [39] and isoniazid [40] represents the most prevalent forms of drug resistance in MTB worldwide, these findings highlight the potential of AMP–antibiotic synergism as a promising strategy to strengthen combination therapies and combat MDR/XDR TB.

Mechanistically, AMPs commonly exert their antibacterial action by disrupting the integrity of the bacterial membrane, which facilitates greater intracellular uptake of antibiotics. This mechanism is particularly beneficial for drugs with intracellular targets, such as rifampicin, amikacin, and azithromycin, whose activity is enhanced by increased intracellular bioavailability [36]. In parallel, many bacteria employ efflux pumps as a major resistance mechanism to reduce intracellular antibiotic concentrations. However, by compromising membrane integrity, AMPs can diminish the efficiency of these efflux systems, ultimately favoring higher intracellular retention of the antibiotic [41]. Consequently, AMPs with strong membrane-permeabilizing activity often display greater synergistic potential when combined with conventional agents.

Although relatively few studies have evaluated the efficiency of synergistic combinations in experimental models, promising evidence supports the use of AMP-based therapies with conventional antibiotics against murine TB and cellular infections. Significant reductions in MTB loads were observed in the lungs, liver, and spleen of mice treated with synergistic combinations of AMPs (e.g., HNP-1) and anti-TB drugs such as isoniazid and rifampicin [42,43]. HNP-1, in particular, appears especially promising due to its human origin, which is likely to confer lower immunogenicity and greater compatibility for clinical applications [44]. On the other hand, AMP-based combinations involving non-human peptides have also shown strong efficacy across in vivo and ex vivo settings. For instance, the bacteriocin AS-48, when combined with ethambutol, was synergistically effective in clearing intracellular MTB from infected macrophages [33]. Similarly, the AMP NZX, when combined with ethambutol, showed a synergistic effect in vivo against murine TB, despite exhibiting only additive interactions in vitro [45]. This discrepancy highlights a crucial consideration in antimicrobial development: in vitro results do not always accurately predict in vivo outcomes, as factors such as bioavailability, host immune responses, and tissue microenvironments may significantly influence therapeutic efficacy.

Emerging drug delivery strategies have been designed to capitalize on AMP–antibiotic synergy. Inhalable dry powder formulations, in particular, are gaining attention as they allow for localized delivery of high drug concentrations directly to the lungs, thereby minimizing systemic exposure [46,47]. For instance, Shao et al. [20] reported that an inhalable formulation combining isoniazid with the AMP D-LAK achieved a fractional inhibitory concentration index (FICI) ranging from 0.25 to 0.38 against clinical MDR MTB isolates, demonstrating strong synergy. Members of the LAK peptide family act by disrupting mycobacterial aggregation through detergent-like effects, which prevent bacterial cell clumping [48]. This mechanism appears advantageous during peptide interaction with the mycobacterial cell wall, as it reduces hydrophobic contacts between the bacterial membrane and thereby enhances antibiotic efficacy [49]. Consistently, D-LAK also exhibited synergistic activity with isoniazid against MDR/XDR MTB isolates while showing no significant toxicity in human monocytic THP-1 cells [48]. Together, these findings suggest that inhalable AMP-antibiotic formulations, particularly those based on LAK peptides, hold considerable promise as safe and effective strategies for treating drug-resistant TB.

Overall, these findings underscore the potential of AMP-based combinatorial therapies to reinvigorate existing TB treatments, particularly in the context of resistant infections. However, despite this hypothesis, several critical challenges hinder the clinical translation of AMPs. One major limitation is their poor stability in vivo due to rapid degradation by endogenous proteases, resulting in a short half-life [50]. In addition, many AMPs exhibit cytotoxicity at therapeutic concentrations, potentially damaging host cells such as erythrocytes and epithelial cells, which restricts their therapeutic window [51].

Nevertheless, synergistic combinations may allow for lower doses of AMPs and antibiotics, thereby reducing toxicity while maintaining efficacy [52]. Efficient delivery also remains a significant hurdle, particularly for pulmonary TB, which requires that therapeutic agents reach infected macrophages and granulomatous lesions. Systemic administration often results in low bioavailability and suboptimal tissue distribution [51]. To address these barriers, recent studies have investigated nanocarrier-based delivery systems and inhalable formulations to enhance AMP stability, reduce toxicity, and promote targeted release at the site of infection [34,37,53]. Although these innovative solutions are promising, rigorous preclinical evaluation is still needed to establish their safety and efficacy prior to clinical implementation.

**Table 1:** Synergistic interactions between AMPs and conventional anti-TB drugs in preclinical studies.

AMP/Antibiotic Combination	Concentrations Used	*Mycobacterium* Strains	FICI	Ref.
HHC-8/Rifampicin MM-10/Rifampicin	1.05 µg/mL (HHC-8 or MM-10) + 0.625 µg/mL (Rifampicin)	*M. smegmatis*	0.09–0.47	[36]
AS-48/Ethambutol	2 µg/mL (each)	*M. tuberculosis* H37Rv	0.09	[32]
M(LLKK_2_)M/Rifampicin	3.91–15.6 mgL^−1^ (M(LLKK_2_)M) + 0.00025–1.95 mgL^−1^ (Rifampicin)	*M. tuberculosis*, *M. bovis*, and *M. smegmatis*	0.56, 0.5, and 0.5	[37]
D-LAK120/Isoniazid	32 µM (each)	*M. tuberculosis*	0.25–0.38	[33]
HNP-1/Isoniazid and rifampicin	6.0–0.1 µg/mL (HNP-1) + 0.3–0.02 µg/mL (each antibiotic)	*M. tuberculosis* H37Rv	0.18–0.25	[41]
NZ2114/Isoniazid and ethambutol	0.88–110 µg/mL (NZ2114), 0.03–2 µg/mL (Isoniazid), 0.06–4 µg/mL (Ethambutol)	*M. bovis* and *M. abscessus*	0.32–0.40	[52]
D-hLF 1-11/Rifampicin	25 µg/mL (D-hLF 1-11) and 0.031 µg/mL (Rifampicin)	*M. tuberculosis* H37Rv	0.312	[53]
NZX/Ethambutol	0.88–110 µg/mL (NZX) + 0.06–4 µg/mL (Ethambutol)	*M. tuberculosis* H37Rv	0.75	[44]

## 4. Designing Synthetic AMPs Against MTB

AMPs are short, naturally occurring or designed peptide sequences that demonstrate antimicrobial properties, often acting as a defensive mechanism for various organisms [54,55,56,57]. These peptides generally contain 10–50 amino acid sequences, exhibiting broad-spectrum activity against bacteria, fungi, viruses, and cancer cells [58]. Their mechanism of action predominantly involves the disruption of target cell membranes, frequently by forming pores or disrupting membrane integrity. They can also interfere with intracellular activities, such as protein synthesis and DNA replication [58,59,60].

AMPs have attracted considerable attention for MTB treatment because of their distinctive ability to target the bacterial membrane with high selectivity. MTB is a notorious infectious agent due to its resilient and complex cell wall, primarily composed of mycolic acid, which makes it resistant to many existing antibiotics. This barrier acts as a safeguard for MTB against environmental stresses, host immune response, and antimicrobial drugs. As a result, TB treatment is becoming challenging due to the emergence of MDR and XDR strains of MTB [4,55,58,60,61,62,63].

Given these challenges, AMP synthesis offers a promising alternative or complement to existing antibiotics. Their capability to target and disrupt bacterial membranes, especially in the lipid-rich components that define the MTB outer layer, provides a unique mechanism of action that may bypass normal resistance mechanisms that affect standard antibiotics [59,60,64,65]. Furthermore, AMPs can be engineered through rational design to enhance their potency, specificity, and stability, making them particularly appealing for the treatment of MDR TB. By designing AMPs with optimized sequences of amino acids that specifically target the individual features of the MTB cell wall, researchers aim to design more cost-effective, targeted solutions to combat TB and decrease the chances of resistance development [60,65,66,67].

AMPs are promising new-generation antimicrobial drugs due to their distinctive features. However, their clinical use is limited by several challenges, including low specificity, poor stability under specific physiological conditions, hemolytic and toxicity side effects, higher production cost, and technical issues, as well as proteolytic degradation [67,68,69]. Therefore, overcoming these challenges has become a key focus of advancement in high-performance AMPs [67]. The design of AMPs is a complex process that involves identifying appropriate amino acid sequences, optimizing their physiological properties, and evaluating their activity against targeted microorganisms [67,69,70,71,72]. Moreover, the amphipathic nature of AMPs, defined by the spatial segregation of hydrophilic and hydrophobic regions in their molecular structures, is essential for their antimicrobial activity. This amphipathicity facilitates penetration into microbial membranes while retaining solubility in aqueous areas. A notable characteristic of AMPs is their brevity sequence, generally consisting of 10–15 amino acid residues. Maintaining a sequence length of less than 50 amino acids provides benefits such as economical synthesis, improved stability, and reduced toxicity, without interruption of activity [67,70,73,74]. AMPs also have a wide range of amino acid compositions, which makes it easier to develop peptides with specialized properties, such as better stability and a stronger affinity for certain targets. Many AMPs are resistant to proteolytic degradation, allowing them to continue functioning in the presence of enzymes that would normally break down peptides or antibiotics. This trait enables them to persist longer in the host and better cope with challenging environments [67,75]. Furthermore, AMPs also demonstrated low selection for mammalian cells, which is crucial for reducing host damage while targeting infections. Understanding the physicochemical features of AMPs can help to overcome the issue related to their design for medicinal applications. Researchers persist in investigating and optimizing these features to develop novel AMPs for diverse therapeutic applications [70]. Rational optimization of these essential physicochemical properties via diverse design strategies, including sequence alterations, conformational restrictions, and lipidation, can enhance the drug-like characteristics and therapeutic potential of AMPs [72,76].

Rational drug design is a methodology for developing novel drugs that depends on a detailed understanding of the microorganism’s biology. This strategy focuses on a thorough examination of the biological target, whether it is a protein, enzyme, receptor, or, in the context of AMPs, a bacterial membrane or other relevant structures. This extensive knowledge facilitates the development of drugs that precisely engage with the target to efficaciously address the infections [77,78,79,80,81].

Furthermore, rational design enables the precise modification of AMPs, optimizing crucial properties such as potency, stability, and selectivity, while reducing toxicity to host cells [78,82]. Through precise engineering, peptides can be customized to enhance their binding affinity to particular components of the MTB lipid-rich cell wall, thereby enhancing their ability to penetrate and disrupt the cell wall membrane [83]. Additionally, computational methods help identify the most promising peptide sequences, thereby enhancing efficacy and reducing uncertain effects. This targeted design strategy enhances the therapeutic potential of AMPs, providing a more selective treatment option, particularly in light of the escalating antibiotic resistance [77,81,82,83,84,85,86].

### 4.1. Peptide Optimization

Computational tools, such as in silico prediction algorithms, molecular docking, and molecular dynamics (MD) simulations, are powerful approaches for optimizing peptide sequences towards specific targets. These strategies provide insights into peptide structure, interaction with targeted membrane or proteins, and stability, without the necessity for longer experimental work [84,87]. These optimization techniques have been successfully employed to design novel AMPs with higher specificity and efficacy against MTB targets like NADH-dependent enoyl-acyl carrier protein reductase [84]. Furthermore, pharmacokinetic screening combined with molecular docking and MD simulations has identified promising AMP mutants as potent inhibitors of multiple MTB enzymes and proteins [88]. Advancements in computational resources and tools for AMPs, including predictive tools and databases, are enhancing the development of novel AMPs to combat antibiotic resistance [89].

### 4.2. Molecular Docking

Molecular docking is a computational technique used to predict the optimal binding configuration of peptides to their targeted protein or membrane. The objectives are to simulate the interaction of a peptide with its targets and provide valuable information on the strength and nature of this interaction via bonding and spaces [90,91,92]. In the case of MTB, molecular docking can be used to predict how AMPs bind to lipid-rich components of the MTB membrane or specific proteins that are essential for bacterial growth and survival. Studies using molecular docking have helped identify and optimize peptides that interact with MTB structural characteristics, such as mycolic acid. For instance, peptides that interact with these lipids can disrupt the membrane, leading to bacterial inhibition [92,93,94].

The optimization of peptides through molecular docking begins with peptide preparation, where the peptide, functioning as the ligand, is carefully refined to ensure it adopts the proper conformation for docking. This process minimizes the energy and structural modifications required to identify peptide stability and biologically relevant interactions with targets. The next step is receptor preparation, which involves target optimization; the target, such as the bacterial cell membrane or protein, is also prepared, typically by refining the 3D structure. After preparing both the peptide and the target receptor, the docking simulation is executed. The peptide is then docked into the receptor using various scoring functions that evaluate the compatibility strength, stability, and quality of the interaction. The docking tools provide multiple binding confirmations, and the best one is selected based on the binding energy and whether it would fit the targeted receptor [88,90,91,92,93,94].

Once a favorable binding pose is identified using molecular docking, rational modification of the peptide sequence can be executed to enhance its activity and stability. Amino acid substitution is a common approach wherein a specific residue is replaced to optimize target interaction. For example, replacing a hydrophobic residue with a hydrophilic one can improve binding to the polar region of a bacterial membrane or a targeted receptor. Increasing the overall positive charge improves electrostatic attraction to a negatively charged microbial surface, thus promoting membrane disruption. The length of the peptide is another main factor; shorter sequences generally increase synthesis and reduce immunogenicity, whereas longer peptides may provide increased functional efficacy and greater target selectivity. Structural changes, such as cyclization or the incorporation of non-natural amino acids like D-amino acids, can enhance proteolytic stability and resistance to enzyme degradation, thereby prolonging the peptide’s lifespan in biological environments. These rational design approaches, informed by docking insights, open the systematic optimization of peptides to enhance the anti-TB activity and therapeutic potential [76,79,95,96,97,98,99].

Peptide docking with membranes or proteins poses significant computational challenges due to the size and complexity of the systems involved. Docking studies often require substantial computational resources, which makes them less accessible to researchers with limited computing capacity. Here, we provide an overview of typical requirements, limitations, and practical approaches to make peptide docking more feasible. One of the main requirements in peptide–protein docking is system size and parameterization. Peptide docking typically involves large molecular systems, generally requiring either all-atom or coarse-grained models of the protein, ligand, and, in the case of membrane-associated peptides, the lipid bilayer. Parameterization typically relies on force fields compatible with both peptides and membranes, such as CHARMM and AMBER for all-atom models, or MARTINI for coarse-grained simulations. A significant challenge is the accurate parameterization of these systems, which is essential for reliably depicting peptide–protein and peptide–membrane interactions. The choice of force field plays a crucial role in predicting binding modes and peptide dynamics. However, the computational complexity increases with the use of more precise force fields, requiring expert knowledge for proper implementation. In addition, parameterization can be time-consuming and prone to errors, often requiring extensive refinement to ensure that all system components are accurately represented [100,101,102].

Run time is another major constraint in peptide docking, particularly for large or complex systems. Standard docking simulations can provide insights into binding affinity and interaction modes, but they are computationally expensive and often require a substantial amount of time to complete. In practice, researchers lacking access to high-performance computing facilities may face significant challenges in conducting such protocols efficiently, creating bottlenecks in their research [102,103]. Another limitation is the high level of computational power needed to handle large systems and long simulations. Typically, docking simulations require access to multicore CPUs and GPUs, which are not universally available. For this reason, access to supercomputer clusters or cloud-based services (e.g., Google Colab, PARAM Shivay at IIT BHU, or the GITAM Supercomputer) may be required to perform such simulations.

### 4.3. Molecular Dynamics

MD simulations are used to investigate the behavior of peptides in a dynamic environment, providing insights into their structural stability and interaction with the bacterial membrane or proteins over time. In contrast to molecular docking, a static approach, MD simulation involves running simulations of peptides within a model of the targeted membrane or environment, allowing the observation of molecular motions and conformational changes [104,105].

MD simulations provide a detailed overview of how peptide behavior is affected in the presence of a bacterial cell membrane, including its ability to penetrate the lipid bilayer. For MTB, characterized by a unique and complex cell wall structure, MD simulations can reveal the interaction between the peptides and mycolic acid and lipids within the membrane. The simulation can also identify whether the peptide has conformational changes that may affect its function or if it could destabilize the membrane through pore formation or membrane disruption [105,106,107,108].

Docking predicts the binding mode, but MD provides insights into peptide stability and flexibility in the bound state, as well as conformational changes that could influence functionality [109,110,111,112]. MD can also replicate peptide behavior in more authentic solvated environments, including water, lipid membranes, and ions, thereby allowing the observation of peptide functionality within the biological surface in which it operates, in contrast to the static conditions of docking [109,110,113]. For peptides targeting *Mycobacterium tuberculosis*‘s bacterial membranes, MD enables simulation of membrane penetration and disruption, offering an evaluation of peptide efficacy in destabilizing the bilayer and its overall impact on membrane dynamics [109,110,113]. Moreover, MD allows for the identification of peptide stability under physiological settings, evaluating whether interactions are maintained or modified under diverse conditions such as temperature and pressure, thereby guiding the optimization of peptides for improved stability and functionality [110,114,115,116].

For instance, Stephanie et al. [117] utilized the molecular docking and MD simulations to optimize a cyclic peptide as an inhibitor of MTB transcription. They started with molecular docking to predict the best binding conformation of the cyclic peptide, which was used as the starting point for MD, targeting the Rifampicin binding site of MTB RNA polymerase (RNAP) using AutoDock Crankpep. Two types of MD simulations were performed using the CHARMM force field within GROMACS: an unrestrained simulation to investigate the peptide’s natural conformational space, and an NMR-restrained simulation found by interpreting distances from NOESY spectra and dihedral angles based on *J*-coupling constants. The constrained simulation demonstrated a predominant and stable conformer exhibiting a BI-turn motif at the Cys1-Leu2-Tyr3-His4 segment, stabilized by intramolecular hydrogen bonding in accordance with NMR temperature coefficient data. This integrative methodology enables the optimization of peptide structure for a biologically pertinent and stable conformation, enhancing its potential as a transcriptional inhibitor [117].

Another study revealed insights into molecular docking and MD simulations. Savintseva et al. [118] conducted a conformational dynamics and stability study of a bilayer formed by mycolic acid from the MTB outer membrane. Peptides targeting the lipid outer membrane of MTB were optimized using a combined approach integrating molecular docking and MD simulations. By docking the peptide and the bilayer mycolic acid, a high binding affinity was identified, and then subjected to the MD in a solvated environment to evaluate their conformational stability, membrane penetration behavior, and potential to disrupt the bilayer integrity. This integrative strategy enabled the selection of a peptide with potential anti-TB properties.

### 4.4. In Silico Prediction Algorithms

Upon MD simulations confirming the stability and membrane interaction potential of selected peptides, in silico tools can further enhance the design process by predicting biological activity and directing sequence modifications. In silico prediction algorithms are the computational methods that simulate and predict the biological action of peptides prior to their laboratory synthesis. These methods use machine learning, bioinformatics methods, and statistical models to evaluate large datasets of peptide sequences and their experimentally known activities. These algorithms predict peptides with potent antimicrobial action, stability, and selectivity against targeted microorganisms, such as MTB, by analyzing patterns of amino acid composition, structural characteristics, and physicochemical properties [119,120].

Sequence-based prediction: A fundamental approach uses the main sequences of peptides. Algorithms identify motif or residue patterns linked to antimicrobial actions. Amino acid frequency, net charge, hydrophobicity, amphipathicity, and sequence length are examined. Cationic residues, such as lysine and arginine, are typically essential contacts, whereas hydrophobic residues enhance penetration into the bacterial membrane. Prediction algorithms can evaluate thousands of peptide sequences based on these properties, allowing them to choose a potential peptide for further evaluation and optimization [73,121,122,123].

Structure-based prediction: Some algorithms incorporate predicted or modeled secondary and tertiary structures. The peptide’s folding, helicity, and amphipathic surface can significantly affect their activity. Algorithms can simulate peptide membrane interactions or predict binding to certain bacterial components, helping to optimize sequences that enhance efficiency while minimizing toxicity to host cells [124].

Database integration: Various in silico tools depend on curated antimicrobial peptide databases, such as APD3, CAMP, and DRAMP. By comparing a new peptide sequence to known active peptides, the algorithms can predict the activity score, potential off-target interaction, and toxicity properties. This significantly reduces the number of experimental peptides, saving time and resources [119,120,125,126].

Optimization of peptides’ properties: In silico prediction is not only used to identify potent active peptides but also to optimize them. Algorithms can suggest sequence modifications to enhance activity, stability, solubility, or resistance to proteolytic degradation. For instance, substituting determined amino acids might enhance membrane affinity and reduce hemolytic activity while maintaining antimicrobial activity [123,127,128,129].

### 4.5. MIC Assays and Functional Characterization

Following the computational optimization, the antimicrobial efficacy of the peptide against MTB is experimentally evaluated using the MIC assay, which determines the lowest concentration of peptide capable of inhibiting bacterial growth. For MTB, these assays are commonly performed using broth microdilution, frequently incorporating surfactants to enhance peptide penetration through the bacterium’s lipid-rich complex cell wall [79]. Complementary minimum bactericidal concentration (MBC) evaluations differentiate between bacteriostatic and bactericidal activity. These results serve as a basis for iterative optimization, directing modification in peptide sequence, charge, hydrophobicity, or structural characteristics to enhance antimicrobial potency [96]. To understand the mechanism of action, peptides undergo membrane disruption assays, such as propidium iodide uptake and calcein leakage from liposomes mimicking the MTB cell membrane, providing direct evidence of membrane permeabilization [130].

Time-kill kinetics provide insights into the speed and extent of bacterial inhibition. At the same time, intracellular activity studies in infected macrophage models, using CFU counts or fluorescent reporter strains, confirm the ability of peptides to penetrate host cells and inhibit intracellular MTB. Synergistic studies with conventional anti-TB drugs can reveal additional or enhanced effects, especially relevant for MDR and XDR strains [127,131]. For the host cell integrity, peptides are evaluated for cytotoxicity against mammalian cell lines and hemolytic activity against human red blood cells. Ultimately, stability studies performed under physiological parameters and in the presence of proteases confirm that the chosen peptides retain their structural integrity and antibacterial efficiency. The MIC investigation and functional characterization give an exhaustive evaluation of peptide efficiency, mechanism of action, and anti-TB effect, confirming predictions from computational docking and MD simulations, and leading to further optimization for anti-TB applications [96,130,131,132,133].

**Figure 1:**
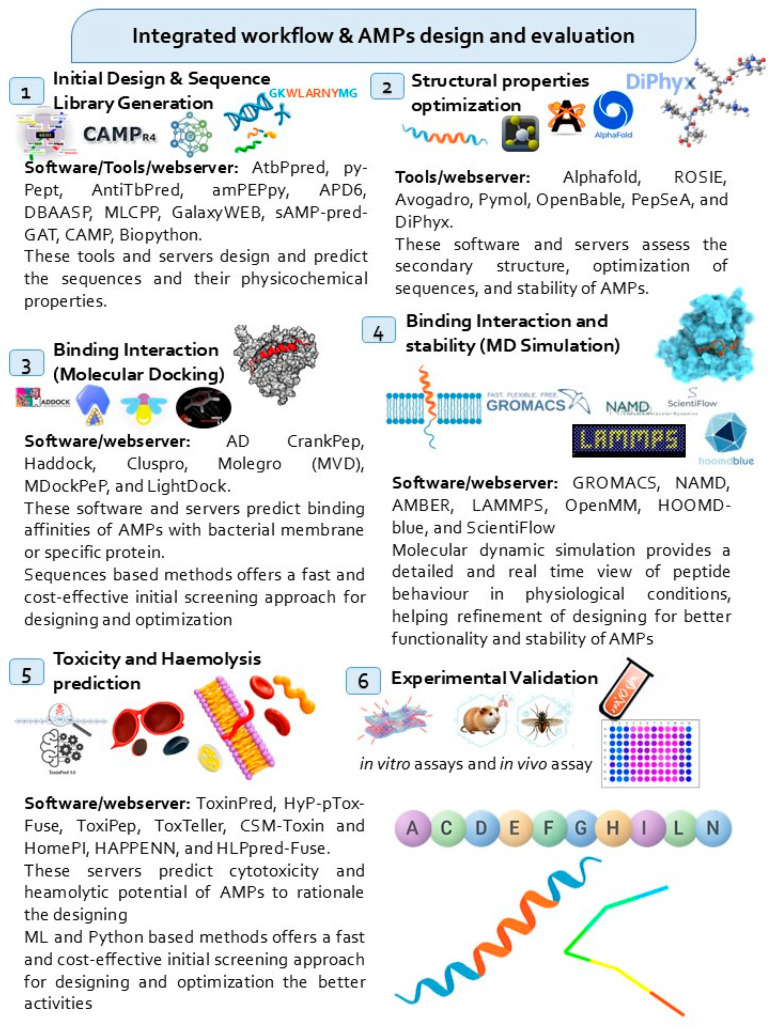
An integrated and accessible workflow for the design and evaluation of antimicrobial peptides (AMPs), spanning from sequence library generation to experimental validation, using freely accessible tools, software, and web servers.

The tools recommended for designing synthetic peptides are mostly chosen based on their efficiency, dependability, and availability. In the initial design phase, tools such as AntitbPred, PyPept, amPEPpy, APD6, CAMP, GalaxyWEB, and sAMPpred-GAT help design and predict AMPs sequences based on their physicochemical properties [119,120,134,135,136,137]. These techniques are essential for preliminary peptide screening and are freely accessible, making them cost-effective options for researchers. In optimizing structural properties, tools such as AlphaFold webserver, ROSIE webserver, Avogadro 1.2.0, PyMOL 3.1, and DiPhyx (cloud based) are most widely used to facilitate secondary structure prediction and stability analysis, ensuring the peptides maintain their functional integrity [138,139,140]. Molecular docking methods, software, and online servers, such as AutoDock, CrankPep, Haddock, Cluspro, and Lightdock, predict the binding interaction and affinity of peptides with bacterial cell membranes or proteins, providing rapid and cost-effective preliminary screening. GROMACS, NAMD, LAMMPS, HOOMD-blue, and OpenMM 8.3.1 software provide MD simulations for predicting AMP stability and interaction under physiological conditions, giving real-time insights into peptide behavior [141,142,143,144]. To further ensure the safety of AMPs, toxicity and hemolysis prediction methods, such as ToxinPred, Hyp-pToxFuse, and HemoPI2.0, as well as HAPPENN and HLPpred-Fuse, are crucial for identifying potential adverse effects before experimental validation protocols [121,145]. These tools are also available for free use. Finally, the last step to validate the experimentally through in vitro and in vivo assays, is necessary to confirm the peptide efficacy, guided by in silico data. These tools and software are recommended for their reliability, ease of access, and capacity to optimize the AMP design process, with various options being open source or freely available to academic personnel (Figure 1).

Recent advancements in the design and evaluation of synthetic AMPs against MTB have exhibited significant laboratory successes. Computational strategies, including docking, MD simulations, and in silico prediction algorithms, have facilitated the systematic selection and optimization of peptide sequences targeting MTB cell wall and intracellular targets, like RNA polymerase *RpoB*. Validated optimized peptides demonstrate the ability to selectively rupture bacterial membranes, penetrate host macrophages, and maintain activity against MDR and XDR strains, as confirmed by MIC, MBC, and intracellular assays. Moreover, experimental studies have confirmed that several peptides maintain stability in a physiological environment, demonstrate low cytotoxicity, and can act synergistically with anti-TB drugs, highlighting their potential as next-generation therapeutics (Table 2).

Despite these promising advancements, several significant challenges remain. The in vivo stability of synthetic AMPs is limited due to proteolytic degradation, particularly in linear peptides, which can reduce half-life and therapeutic efficiency. Bioavailability is often diminished by poor absorption and rapid systemic clearance, hindering effective delivery to the lungs, the primary site of MTB. Additionally, balancing antimicrobial potency with host safety remains challenging, as highly cationic or hydrophobic peptides may induce hemolysis or cytotoxicity. Resistance development, although less common than with existing antibiotics, is also a concern, highlighting the emergence of precise sequence optimization. Ultimately, high synthesis costs, particularly for cyclized or chemically modified peptides, present a substantial obstacle to large-scale production and clinical application.

## 5. Vitamin D, Cathelicidins, and Tuberculosis

Vitamin D deficiency, one of the most prevalent micronutrient deficiencies worldwide, has been strongly associated with susceptibility to TB and poorer treatment outcomes [155]. According to global estimates, more than half of the world’s population is at risk, and vitamin D deficiency—particularly in immunocompromised or malnourished patients—has been strongly associated with susceptibility to tuberculosis and poorer treatment outcomes [156]. Multiple studies suggest that administering high-dose vitamin D supplementation to patients with this micronutrient deficiency during treatment for MTB reduces the time to sputum culture conversion and accelerates clinical and radiographic improvements [157,158,159]. Therefore, this suggests that supplementing vitamin D alongside conventional TB therapy can be beneficial, highlighting its potential role in adjunct therapies.

Vitamin D is a steroid hormone that can be obtained through diet, vitamin supplements, or synthesized in human skin from 7-dehydrocholesterol when exposed to UV light [160]. To become functional, it requires two stages of activation. First, it is hydroxylated into 25-hydroxyvitamin D_3_ (25D_3_) in the liver by 25-hydroxylase. Then the compound is transported to the kidneys, where the second hydroxylation is performed by the 1-α-hydroxylase, resulting in the formation of an active metabolite, 1,25-dihydroxyvitamin D_3_ (1,25D_3_) [161].

Furthermore, the active form then circulates in the bloodstream, bound to vitamin D-binding protein and transported to the nuclear vitamin D receptor (VDR), which belongs to the superfamily of nuclear receptors, along with other steroid hormone receptors [162]. The 1,25D_3_ binds to the VDR and is transported to the nucleus, where it interacts with the retinoid X receptor, forming a heterodimer that enables interaction between the transcriptional machinery and the promoters of 1,25D_3_ target genes involved in various processes, including the host immune response [162,163,164]. Additionally, circulating levels of 1,25D_3_ need to be carefully regulated to prevent excessive VDR signaling, which is inactivated by the enzyme 24-hydroxylase [160]

Regarding the immune system, some immune cells, such as monocytes and macrophages, can also express both activation (25- and 1-α-hydroxylase) and inactivation (24-hydroxylase) enzymes, regulating 1,25D_3_ levels at the infection site [160]. This allows local innate immune stimuli to activate VDR signaling, improving host defense mechanisms, through the induction of interferon-gamma (IFN-γ) production in host cells against intracellular pathogens and the stimulation of cathelicidin LL-37 production, for example, playing a critical role in bacterial clearance [164,165]. During MTB infection, 1,25D_3_ induces antimycobacterial activity, for example, through the expression of LL-37 by lung epithelial cells, macrophages, and neutrophils, promoting phagolysosome fusion, reactive oxygen species generation, and autophagy, while modulating the immune response through cytokine expression, thereby aiding in the elimination of MTB [26,166,167].

Cathelicidins, particularly human LL-37, exert both immunomodulatory and direct antimicrobial functions through well-defined biochemical mechanisms. LL-37 displays a strong affinity for anionic phospholipid-rich bacterial membranes, where its amphipathic α-helical structure enables insertion and pore formation, thereby disrupting membrane integrity and collapsing the electrochemical gradient [66]. During MTB infection, LL-37 also modulates macrophage responses by downregulating pro-inflammatory cytokines, such as TNF-α and IL-17, while upregulating IL-10 and TGF-β, thereby limiting tissue damage without compromising bactericidal activity. In vitro studies have demonstrated a dose-dependent reduction in mycobacterial burden [28]. Moreover, LL-37 can localize to host mitochondria, where it induces the release of apoptogenic factors such as apoptosis-inducing factor, highlighting its ability to perturb mitochondrial membrane permeability. Biophysical assays with liposomes mimicking mitochondrial lipid composition further confirm a direct structural destabilization effect [168].

In this context, vitamin D supplementation can lead to immunomodulation, providing an alternative for the prevention or treatment of MTB infection. Arifin et al. [169] demonstrated that oral supplementation with 10.000 IU of vitamin D_3_ per day for 8 weeks had the potential to elevate the levels of TLR2 and TLR4 during the infectious process. Moreover, in another study, patients with a low baseline serum level of vitamin D exhibited an increase in IFN-γ production induced by MTB after an intramuscular injection of 400.000 IU of vitamin D_3_, illustrating the immunomodulatory effects of supplementation, which aid treatment in the initial stages of disease [170].

Furthermore, poly(lactide) nanoparticles with calcitriol (1,25(OH)_2_D_3_) had a bacteriostatic effect and reduced lung consolidation in MTB-infected mice [171,172,173]. In another study, encapsulation of calcitriol and calcifediol (25(OH)D_3_) with poly(ε-caprolactone) nanoparticles enhanced LL-37 production in THP-1 macrophages compared to their free forms, which is possibly related to an increase in macrophage uptake and molecular stability, favoring the activation of the VDR-CAMP pathway [174]. Also, small vitamin D-based molecules (D-VITylation) covalently linked to therapeutic peptides and proteins have already demonstrated an increase in half-life and bioavailability, favoring peptide drug development [175].

## 6. AMPs and the Tuberculosis Vaccine Landscape

Drug-resistant MTB strains are becoming a public health threat due to their low cure rates, high mortality rates, and expensive healthcare costs. In this scenario, vaccines have become a pillar to control the TB epidemic and the spread of drug-resistant MTB strains [176].

Bacillus Calmette-Guérin (BCG), first used in 1921, is an attenuated *M. bovis* vaccine. It is also the most widely used preventive TB treatment in the world [177]. Evidence suggests that BCG vaccination offers protection to newborns and young children against severe forms of TB, such as pulmonary and disseminated forms. However, its efficacy has varied, depending on geographical factors and prior exposure to environmental mycobacteria [178]. To investigate whether revaccination would address the limitations of BCG, a randomized, double-blind, placebo-controlled Phase 2b trial was conducted in South Africa. Although limited effectiveness against primary MTB infection was observed, revaccination demonstrated significant protection against sustained de novo infection, resulting in prolonged IGRA conversion [179].

MTB has immune evasion strategies, such as delaying adaptive responses and inhibiting CD4^+^ T cell activation, which represent major obstacles to the development of effective vaccines. Vaccines capable of overcoming immune evasion, inducing a long-lasting immunity, and resisting reinfection and chronic exposure are needed [180].

Recent advances in TB vaccine development in clinical trials can be classified into four categories: whole-cell mycobacterial vaccines, viral vector vaccines, mRNA-based vaccines, and subunit vaccines. Whole-cell approaches, such as BCG, can be divided into live-attenuated vaccines derived from genetically modified MTB (e.g., MTBVAC) and killed mycobacteria, such as *Mycobacterium vaccae.* Some of their benefits include the presentation of a large array of antigens—for example, proteins and glycolipids—which stimulate a more diverse immune response [176].

Viral vector vaccine platforms use engineered viruses to encode MTB’s antigens. This strategy enables the activation of an immune response to this specific pathogen’s antigen [176]. Some of its diverse advantages are the ability to carry large antigen sequences and the induction of both humoral and cellular immunity. They also have the benefit of not requiring adjuvants. However, disadvantages include contraindications for immunocompromised populations and efficacy limitations because of pre-existing antibodies against the viral vector. Some examples of recent TB viral vector vaccine candidates are MVA85A, AdHu5Ag85A, and TB/Flu-05E [181].

Additionally, mRNA vaccines use single-stranded RNA molecules that encode TB proteins. These molecules are translated by host ribosomal machinery into the encoded antigen [182]. This platform is easier to manufacture, and it can induce both humoral and cell-mediated immune responses. Nevertheless, it has some limitations: inefficiency of in vivo delivery, intrinsic instability, and high innate immunogenicity. ID91 is an example of an mRNA TB vaccine candidate [183].

Lastly, subunit vaccines are developed to activate the immune response to pathogen-specific targets (e.g., polysaccharides, proteins, or small peptides). Their main challenge is selecting an antigen that optimizes the immune response in a diverse population. With the right antigen selected, the vaccination regimen offers greater dose control and a more favorable safety profile for immunocompromised populations. An important characteristic of this group is the requirement for adjuvants in the formulation due to the absence of pathogen-associated microbial patterns (PAMPs). Two examples of subunit vaccine candidates are H4/IC31 and M72/AS01 [184].

A subtype of subunit vaccines, peptide-based vaccines use short, pathogen-specific peptides as their pillars. A selective pathogen antigen avoids cross-reactivity with human proteins. They have gained attention due to their safety, low production cost, precise characterization, lack of pathogenic sequences, manufacturability, and ease of storage at room temperature. At the same time, their low immunogenicity remains a challenge, particularly in single-epitope formulations. They typically trigger the following response: dendritic cells present these peptides to active naive T cells, which migrate to infection sites and mediate the immune reaction [185].

For better selection of antigenic epitopes capable of eliciting B-cell and T-cell responses, and for the optimization of vaccine candidates, reverse vaccinology and immunoinformatics can be powerful tools. A recent example of the application of these approaches in designing a peptide-based vaccine is the study by Mboowa et al. [186]. They employed an in silico approach to design a multi-epitope vaccine targeting the PE_PGRS16 protein, a conserved virulence factor of MTB and *M. bovis*, as well as epitopes for B-cells, cytotoxic T lymphocytes, and helper T lymphocytes. This study demonstrates the potential of reverse vaccinology for global vaccine deployment [186].

**Figure 2:**
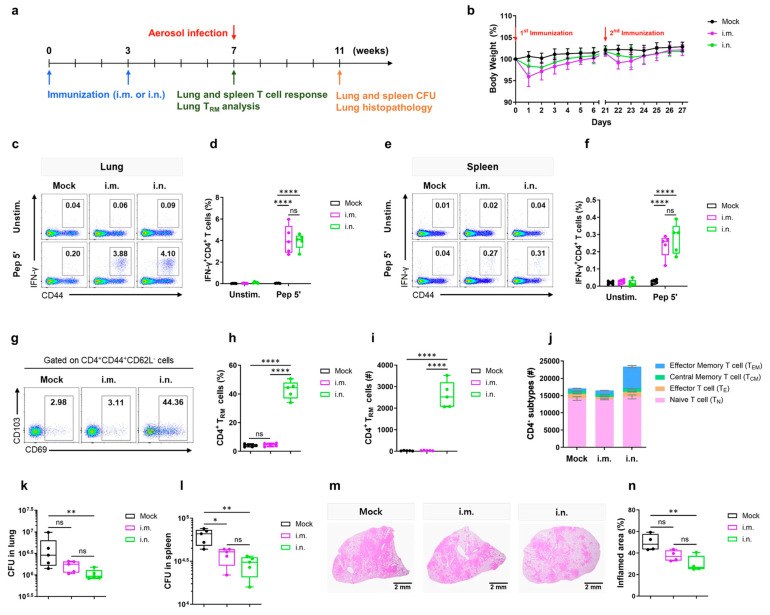
Experimental design and immune responses induced by the Nexavant-formulated peptide vaccine in mice. (**a**) Vaccination schedule: C57BL/6 mice were immunized intramuscularly (i.m.) or intranasally (i.n.) twice at 3-week intervals, followed by immune response analysis or aerosol challenge with *M. tuberculosis* H37Rv. (**b**) Body weight changes after immunization. (**c**–**f**) IFN-γ^+^CD4^+^ T-cell responses in lung and spleen. (**g**–**i**) Induction of lungs’ resident memory CD4^+^ T cells (TRM). (**j**) Distribution of CD4^+^ T-cell subsets in the lung. (**k**–**n**) Post-challenge bacterial loads in lungs and spleen, and lung histopathology showing reduced inflammation in immunized animals. Reprinted from Ko et al. [188], under the terms of the Creative Commons Attribution-NonCommercial-NoDerivatives 4.0 International License. * Statistical significance is indicated as follows: ns, not significant; *, *p* < 0.05; **, *p* < 0.01; ****, *p* < 0.0001.

Ho Hwang and colleagues’ study is another example that applies immunoinformatics to design a subunit vaccine candidate against MTB [187]. This formulation included epitopes for B cells, cytotoxic T cells, and helper T lymphocytes. They used docking, molecular dynamics simulations, essential dynamics analysis, and in silico cloning to predict its immunogenic profile and validate the vaccine’s efficacy. Their analysis suggests this candidate has strong potential against TB by inducing specific immune responses [187]. A recent and promising vaccine platform is the intranasal Nexavant-formulated peptide vaccine (Figure 2). This formulation was designed by elongating the CD4^+^ T cell epitopes from MTB antigens ESAT-6, CFP-10, and HspX, and adjuvanted with Nexavant, a TLR3 agonist. This research demonstrated the induction of lungs’ resident memory T cells and protection against MTB infection, as shown by lung histopathology of mice infected and immunized with the vaccine candidate. These findings also provide a basis for designing an inhalable TB vaccine using peptides [188].

In order to design an effective peptide-based vaccine, it is important to identify potential peptides with diverse MHC binding affinities, predict the most promising immunodominant lymphocyte epitopes, and assess the immunogenicity, antigenicity, and toxicity of the chosen epitope. Additionally, the selection of a suitable linker (flexible, rigid, or cleavable) and adjuvant peptides (TLR agonists and helper peptides) is crucial for an optimal response. Whereas TLRs are critical in both innate and adaptive immune responses (especially TLR2-, TLR4- and TLR9-mediated responses in TB), a strategy to enhance the immunogenicity of peptide-based vaccines is adding specific agonists to these receptors. Another strategy to improve the efficacy of vaccines is adding peptides to enhance their immune effects [189].

In a study by Jun Tye et al. [190], combinations of the peptides MHC-I (AFPSFAGL), MHC-IIª (EFSELFAAFPSFAGL), and MHC-IIb (EFAYGSFVRTVSLPV) were tested with the CASAC (IFN-γ, poly(I/C), and CpG-ODN) adjuvant in a murine model. The objective of this study was to evaluate the potential of this system to promote cellular immunity against the HspX antigen. Its results showed an increased cytotoxic CD8^+^ T cell activity, a higher Th1 cytokine production (IFN-γ and IL-2), and cellular memory formation. At the same time, these combinations did not induce an intense regulatory T response [190].

A Phase 1, randomized, double-blind clinical trial was conducted in 60 BCG-naive adults to evaluate two doses of the ID93 antigen, administered alone or in combination with the GLA-SE adjuvant. ID93 is a subunit TB vaccine candidate comprising four MTB-antigens: Rv1813, Rv2608, Rv3619, and Rv3620. Meanwhile, the adjuvant GLA-SE is a TLR4 agonist formulated in an oil-in-water nano-emulsion. This vaccine candidate demonstrated a safe profile and induced functional humoral and Th1-type cellular responses (e.g., TNF^+^, IL-2^+^, and IFNγ^−^) [191].

Moreover, a randomized, open-label, Phase I/II clinical trial was conducted with 222 patients to evaluate the safety and immunogenicity of vaccination with H56/IC31 and a cyclooxygenase-2 inhibitor (COX-2i) in pulmonary and extra-pulmonary TB patients. Its formulation is composed of H56/IC31, a fusion protein of Ag85B, ESAT-6, and Rv2660c, and the adjuvant IC31, which is composed of a helper peptide (KLK) and a TLR9 agonist (the oligodeoxynucleotide ODN1a). At the same time, COX-2i is a non-steroidal anti-inflammatory drug used to treat TB symptoms and antitubercular drugs’ side effects. A significant increase in antigen-specific T cell population was observed in the population vaccinated with H56/IC31 and COX-2i [191].

To design an effective TB vaccine, it is essential to understand the pathogenesis of MTB [192]. Normally, the host inhales aerosols containing MTB bacilli, which are phagocytosed by alveolar macrophages. As the infection progresses, these bacilli stop the phagocytosis process and infect antigen-presenting cells, activating diverse innate response pathways. Some of these responses are involved in phagocytosis, vesicle trafficking, and production of inflammatory cytokines [193]. The predominant MTB virulence mechanisms include phagosome membrane disruption and impaired mitochondrial metabolism. These changes trigger type I interferon responses (e.g., IFN-α, IFN-β), suppress TLR2 signaling, and enhance MHC-I antigen presentation. They also promote downstream CD8^+^ T cell activation, autophagy reduction in antigen-presenting cells, eosinophil recruitment, and the suppression of early Th17 responses [194].

On the other hand, cell-mediated immune responses involving CD4^+^ T-helper 1 (Th1) cells and CD8^+^ cytotoxic T lymphocytes are essential for the control of TB, both in acute and latent forms [195]. However, the intracellular infection interferes with the early antigen presentation by antigen-presenting cells, thereby retarding the mobilization of activated lung CD4^+^ T cells (e.g., CD4^+^ T-memory). In the vaccination scenario, it is also possible to observe a delayed antigen presentation by MTB-infected cells. This is due to the accumulation of vaccine-induced memory T cells in the lymph nodes before their mobilization to the lungs. In order to overcome this phenomenon, Khader et al. [196] transferred dendritic cells into vaccinated mice at the time of infection. Their study was successful in accelerating specific CD4^+^ T-cell responses, resulting in improved vaccine-mediated protection [196].

Dendritic cells are crucial in bridging the gap between innate and adaptive immune responses [197]. They produce IL-12p40 via TLR-9, which promotes IFN-γ production by T cells. This protein forms the heterodimers IL-12p70 and IL-23, which are necessary for Th1 differentiation and Th17 cell activity, respectively. In this way, a promising strategy to enhance cell maturation and antigen presentation is targeting dendritic surface markers, such as DEC-205 [198].

AMPs, as components of the innate immune system, have an important role in MTB infection and immunoregulation. Hernández-Pando et al. [199] assessed the immunotherapeutic effects of AMPs in pulmonary TB. This study showed that adenoviruses encoding HβD3 and LL37 reduced the lung bacterial load while inducing the expression of pro-inflammatory cytokines (IFN-γ and TNF-α) [199].

Thus, peptides derived from immune cells have an important role as immunomodulators and antimycobacterial agents. For example, studies using β-defensin HBD-2 have shown reductions in MTB growth in vitro and in vivo, with synergistic effects when combined with isoniazid and rifampicin. Additionally, the AMP granulysin has demonstrated the ability to disrupt bacterial membranes and induce apoptosis in host cells. In addition, lactoferrin has also been shown to reduce the spread of bacilli to the liver and lungs, as well as enhance pro-inflammatory cytokines (e.g., IL-12), thereby promoting Th1 responses [50].

Furthermore, IFN-γ has historically been used as a fundamental biomarker to assess vaccine immunogenicity. Because of that, most TB vaccine studies, including BCG’s assessment, have used measuring IFN-γ as an indicator of efficacy [200]. Despite that, recent evidence suggests that a broader range of immune markers should be considered. These include the presence of surface marker CD153 on CD4^+^ T cells and specific IgA in bronchoalveolar fluid. These findings support the hypothesis that, although Th1 immune responses are important, the immune protection against MTB is not limited to this immune pathway [180].

However, the development of novel vaccines against TB has significant limitations. For example, animal models used in preclinical stages often fail to predict human responses nowadays. Thus, it remains difficult to understand which MTB epitopes are truly presented by infected cells. These limitations can lead to the failure of some vaccine candidates. In this way, the development of better methodologies to safely access infected human cells and detect peptides is needed. Promising technologies in this field are, for example, two-photon intravital microscopy, single-cell transcriptomics, and T cell receptor analysis. Yet, expensive costs and biosafety remain limitations of these techniques [201].

Therefore, vaccination is a key strategy to accomplish the World Health Organization (WHO) goal of TB eradication by 2035. Because of BCG’s limitations, the development of more effective vaccines is indispensable. Advances in bioinformatics and AI bring hope to the discovery of new antigens and the prediction of immune responses. Diverse MTB-antigens, especially virulence factors, have emerged as potential candidates. Examples of these vaccine targets are ESAT-6 (EsxA) and CFP-10 (EsxB). Other strong target candidates for T cell response in acute and latent TB are: TB10.4 (EsxH), Ag85A/B/C, PPE18, Rv1813c, HspX (Rv2031c), PepA, and Rv2626c. All the antigens above have the capability to modulate humoral responses and activate both CD4^+^ and CD8^+^ T cells (especially Th1 cells). Due to these characteristics, they have been incorporated into vaccine candidates in clinical trials, such as M72/AS01E, ID93+GLA-SE, H56/IC31, MTBVAC, and MVA85A [202].

## 7. Delivery Systems

Nanotechnology

Nanotechnology in the context of delivery systems refers to the use of materials and systems at the nanometric scale (generally between 1 and 100 nanometers) to transport and release drugs, genes, proteins, or other therapeutic molecules in a controlled and targeted manner within the body [203]. The complex pathogenesis of MTB, characterized by its ability to evade host immune responses and establish latent infections, together with the emergence of MDR-TB and XDR-TB strains, requires innovative treatment strategies, particularly advanced drug delivery systems. This is where nanotechnology plays a pivotal role, primarily utilizing polymeric nanoparticles, liposomes, and lipid particles for sustained, targeted, and inhalational drug delivery. This approach has demonstrated prolonged release, delivery to macrophages, intracellular activity against MTB, and reduced infection in animal models [204].

MTB is an obligate intracellular pathogen that primarily infects pulmonary alveolar macrophages following inhalation of bacteria-containing droplets [205,206]. Early interactions between MTB and the host innate immune system are crucial in determining the establishment of infection and disease progression [207]. It has evolved sophisticated mechanisms to subvert the host immune response and survive within macrophages, which serve as its primary site of replication. One key mechanism involves the inhibition of phagosome–lysosome fusion, a critical step in the host’s ability to destroy internalized bacteria [208,209]. MTB actively prevents the maturation of phagosomes into phagolysosomes, thereby creating a permissive intracellular environment for its survival and replication [210]. Several studies have shown that MTB phosphoribosyltransferase (MTB PRT) can inhibit autophagy, a cellular process essential for the elimination of intracellular pathogens, by inducing histone hypermethylation at the Atg5 and Atg7 promoters through the activation of p38-MAPK and EHMT2 methyltransferase-dependent pathways [211].

This inhibition of autophagy allows MTB to evade destruction and persist in host cells. In the context of sustained drug release, research has focused on strategies that increase intracellular delivery (to alveolar macrophages) and drug retention, using systems that facilitate sustained release, bioadhesion, and direct pulmonary administration. These strategies include PLG/PLGA nanoparticles and microparticles, liposomes, solid lipid particles, and functionalized formulations (such as lectins) to increase bioavailability and targeting. In vivo studies have shown that surface functionalization (e.g., with lectins) increases the relative bioavailability and prolongs the plasma detection of anti-TB drugs, while encapsulation in biodegradable matrices facilitates sustained release, thereby reducing the dosing frequency. Furthermore, inhalation therapies, including nebulization and dry-powder formulations, are proposed as preferred routes for pulmonary TB treatment, as they enable direct delivery of the drug to the diseased lung tissue and alveolar macrophages. Table 3 summarizes recent advances in drug administration strategies [204,212,213].

On the other hand, peptide-based nanocarriers are a developing strategy with great progress in the preclinical area that combines the use of AMPs, peptide–metal conjugates with lipid, polymeric, inorganic, carbon nanoparticles, as well as self-assembled, peptide-conjugated nanoparticles, to revitalize drug [220], improve stability, and facilitate macrophage delivery and intracellular activity. This approach attain key objectives such as protection against proteolysis and renal clearance—as encapsulation or conjugation protects peptides from enzymatic degradation and prolongs circulation time—targeted delivery to intracellular reservoirs—as size optimization, surface functionalization and inhalation routes allow specific action on alveolar macrophages and granulomas [217,219]—and the potential use in combination therapy—as co-loading of AMPs with conventional anti-TB drugs allows for synergistic effects and reduces required dosages [218,221]. In this way, peptides can be used as nanocarriers that transport drugs, serve as encapsulated bioactive molecules, or act as conjugates associated with nanoparticles. Furthermore, advances from 2020 to the present have demonstrated progress in elucidating mechanisms of action much more clearly (membrane disruption, intracellular targeting, and host-directed immunomodulation), developing macrophage-targeted platforms, and promising in vitro/in vivo proofs of concept (Table 4). However, clinical application remains limited by toxicity, proteolysis, granuloma penetration, and manufacturing and regulatory deficiencies.

### 7.1. Host-Directed Mechanisms

Immunomodulation: MTB also manipulates host cellular stress responses and immunological regulations to ensure its survival [226]. It can alter cell death mechanisms of host macrophages, which are crucial for infectivity and dissemination. Furthermore, MTB can enter a state of dormancy or latency, characterized by reduced metabolic activity, which enables it to survive asymptomatically in the host for decades, contributing to phenotypic drug resistance [227]. This dormant state is particularly challenging for conventional antibiotics, which often target actively growing bacteria. The ability of MTB to reside in lysosome-poor monocyte-derived lung cells during chronic infection further highlights its adaptability and immune evasion strategies.

AMPs not only act directly on pathogens but also play an essential role in modulating the host immune response. These compounds can induce macrophage activation, promoting a more effective response to infections, while also promoting phagosome–lysosome fusion, a critical step in the degradation of intracellular bacilli, and stimulating autophagy processes that contribute to the elimination of persistent bacteria [228]. Such mechanisms are particularly relevant in the case of MTB, since this pathogen has developed strategies to evade lysosomal destruction and survive in intracellular compartments [216,228]. Therefore, the ability of AMPs to restore or enhance these immunological pathways represents a significant advantage in overcoming the limitations of conventional treatments and reducing the risk of bacterial resistance.

Anti-Biofilm Activity: Bacterial biofilms are multilayered cellular communities surrounded by a matrix of extracellular polymeric substances (EPS) that protects the bacteria and confers particular resistance to antimicrobials. This resistance is due to various mechanisms, including limited penetration of antibiotics by the matrix, enzymatic degradation of compounds, the presence of extracellular DNA that stabilizes the structure and reduces drug efficacy, hypoxia in the biofilm core that affects oxygen-dependent antibiotics, and slow growth and metabolic dormancy that diminish the action of agents targeting dividing cells. Furthermore, bacteria can activate efflux pumps to expel antibiotics and employ quorum sensing to upregulate genes associated with resistance and biofilm maintenance. Together, these factors explain the persistence of biofilm-associated infections and highlight the urgency for developing new antimicrobial strategies capable of overcoming these barriers [229]. On the other hand, granulomas are cellular aggregates formed in response to chronic inflammation. They are composed of monocyte-derived macrophages, foam cells, epithelioid cells, and multinucleated giant cells. These cells coordinate immune defense by activating T cells that are capable of inducing apoptosis in intracellular mycobacteria through the action of granzymes and granulysin. The production of cytokines and chemokines, along with mediators such as IFN-γ and TNF-α, recruits B and NK cells and promotes the differentiation of CD4^+^ lymphocytes into T1 cells under the influence of IL-12. Although traditionally considered restrictive structures for the growth of MTB, recent research has shown that granulomas constitute dynamic environments with interrelated microenvironments that, in some cases, act as reservoirs for mycobacteria. Evidence from zebrafish models infected with *Mycobacterium marinum* has shown that monocyte recruitment within the granuloma can even promote bacterial proliferation, highlighting the need to develop therapeutic strategies targeting macrophages or the interior of these structures [53]. In addition to their immunomodulatory effects, AMPs have demonstrated efficacy against mycobacterial biofilms and granuloma-like structures, which constitute physical and biochemical barriers that hinder the action of conventional antibiotics [216,230]. These biofilms confer on MTB a remarkable capacity for persistence and resistance in chronic settings, contributing to therapeutic failure and prolonged treatment. In this context, the action of AMPs can destabilize the extracellular matrix of biofilms, increase permeability, and facilitate greater drug penetration towards the bacteria hosted inside. As a result, not only is therapeutic efficacy improved, but also the likelihood of the emergence of resistant populations is reduced, making AMPs particularly valuable candidates for treating chronic and difficult-to-eradicate mycobacterial infections.

### 7.2. Drug Delivery Systems and Nanocarrier Platforms

#### 7.2.1. Macrophage-Targeted Delivery

Invasion of macrophages by MTB induces the production of granulocyte–macrophage colony-stimulating factor, which activates the expression of the CISH protein through STAT5. This protein promotes the ubiquitination and degradation of the catalytic subunit A of V-ATPase, blocking the recruitment of GTPases and V-ATPases to the phagosome membrane. Additionally, MTB uses the ESX-1 secretion system to damage and permeabilize this membrane, sometimes facilitating its escape into the cytosol. In response, nitric oxide synthase 2, induced by IFN-γ, activates infected macrophages and inhibits intracellular replication of MTB, while the LRG-47 protein complements this action by reinforcing cellular resistance. During this process, macrophages accumulate lipid bodies and become foam macrophages, which play a key role in the establishment, persistence, and dissemination of infection within the granuloma [53]. Another key strategy is to specifically target formulations to alveolar macrophages, which are considered the main reservoir of intracellular MTB. To this end, it has been shown that adjusting the size of nanoparticles within the range of 100–500 nm favors phagocytosis. In parallel, the conjugation of specific ligands, such as mannose, galactose, or antibodies, on the particle surface enhances receptor-mediated uptake [217,222]. Furthermore, optimizing the surface charge towards positive values contributes to intensifying the interaction with the cell membrane and, consequently, improves the internalization of the delivery system [221,222].

#### 7.2.2. pH-Responsive and Stimuli-Responsive Systems

MTB encounters a range of environmental stresses within the host, with acidic pH being a primary cue that triggers widespread transcriptional and metabolic remodeling [231,232]. At mildly acidic pH (e.g., <pH 5.8), MTB ceases replication, a process known as “acid growth arrest”, which contributes to its virulence [233,234]. The molecular mechanisms underlying MTB’s acid resistance involve complex regulatory networks and metabolic shifts. For instance, MTB alters its central carbon metabolism, becoming dependent on lipids for growth in acidic environments. This metabolic rewiring involves reducing glyceraldehyde-3-phosphate dehydrogenase (GAPDH) activity, which diverts glucose catabolism away from glycolysis and toward gluconeogenesis and the glyoxylate shunt, enabling efficient lipid assimilation.

Nanocarriers capable of releasing their cargo in response to specific microenvironmental conditions represent a highly innovative alternative for treating tuberculosis [213,223]. In this sense, pH-responsive systems release their contents into the acidic environment characteristic of phagolysosomes. In contrast, enzyme-sensitive systems utilize the degradation mediated by mycobacterial or host enzymes to induce drug release [223]. In a complementary manner, redox-responsive nanocarriers respond to the reductive conditions of the intracellular environment, offering an additional controlled release mechanism that increases therapeutic specificity and efficacy [223].

#### 7.2.3. Microtechnology

Microtechnology involves the use of materials with diameters generally ranging from 1 to 1000 μm (micrometers). Microparticles are widely used in targeted drug delivery for the treatment of TB, especially in pulmonary administration. These particles can be deposited deep within the lungs, specifically in the bronchi and alveoli, providing improved absorption and therapeutic efficacy. Some notable examples of microparticle applications are detailed below:

PLGA (poly(lactic-co-glycolic acid)) microparticles represent a promising strategy for delivering drugs against TB, thanks to their biodegradable and biocompatible nature and ability to release compounds in a controlled manner at the site of infection [235]. PLGA-encapsulated rifampicin microparticles, administered by inhalation, have been shown to effectively reach alveolar macrophages infected with MTB, thereby improving therapeutic efficacy and reducing systemic toxicity. Furthermore, the safety of PLGA for pulmonary use, together with the development of innovative methods that enable the production of uniformly sized particles, reinforces its potential as a platform for the targeted and sustained release of anti-TB drugs. On the other hand, there are cases, such as that of microencapsulated rifabutin SLNs (solid lipid nanoparticles) with trehalose and mannitol, both excipients approved by the FDA, where an accelerated release of the active ingredient has been observed within the first hour after administration, which favors a rapid availability of the antibiotic at the site of action [236].

### 7.3. Clinical Applications and Translational Studies

#### 7.3.1. Preclinical Development Status

Peptide- and nanocarrier-based systems for the treatment of TB are mostly in preclinical development stages, although several candidates have shown promising results at different stages of experimental evaluation [216,230]. In vitro studies have shown that these systems increase antimicrobial activity against multidrug-resistant MTB strains, highlighting their potential to address one of the main limitations of current therapeutics [216,218]. Furthermore, research on cellular uptake has shown a significant improvement in macrophage internalization and an increase in intracellular drug concentrations, which is critical for combating infections that persist within intracellular compartments [218,228]. These findings have been reinforced by studies in animal models, where a marked reduction in bacterial load has been observed in mice infected with MTB, providing strong preclinical evidence to advance to later stages of development.

#### 7.3.2. Regulatory Considerations

Despite these advances, the translation of peptide-nanocarrier systems into clinical practice faces significant regulatory challenges [213,228]. One of the main challenges lies in conducting a thorough safety assessment; the introduction of new nanomaterials requires complete toxicological studies to rule out long-term adverse effects [237]. In addition, there is a need to establish standardized and reproducible manufacturing methods that guarantee strict and consistent quality control in each production batch [222,237]. Another fundamental aspect is the detailed characterization of the formulations from both the physicochemical and biological perspectives, which includes the precise definition of parameters such as size, surface charge, stability, release kinetics, and cellular interaction profiles [237]. Compliance with these regulatory criteria is an essential requirement for facilitating the transition from the experimental phase to clinical studies in humans.

#### 7.3.3. Clinical Trial Landscape

To date, no peptide- and nanocarrier-based system specifically targeting the treatment of TB has reached the clinical trial stage. However, related technologies applied in other infectious diseases have provided valuable precedents in terms of both regulatory and therapeutic feasibility [222,237]. This includes the development of nanocarriers for the delivery of antivirals and antibacterials in pathologies where the need to overcome biological barriers and improve bioavailability is equally critical. These advances offer previously explored regulatory avenues that could facilitate the design of future clinical studies in TB, while providing evidence on the potential safety and efficacy of peptide- and nanomaterial-based platforms. In this context, the experience accumulated in other therapeutic fields can serve as a basis to accelerate the clinical translation of these technologies towards specific applications against MTB.

### 7.4. Challenges and Limitations

#### 7.4.1. Technical Challenges

Peptide Stability: Despite the protection offered by nanocarriers, peptide stability remains a relevant challenge, especially in oral applications or in contexts requiring prolonged storage. Peptides are highly susceptible to enzymatic degradation and denaturation processes, which can compromise their bioavailability and biological activity. To counteract this problem, several strategies have been proposed, including chemical modification of the peptide sequence to increase their resistance to proteases, the inclusion of enzyme inhibitors in formulations, and the optimization of preparation and storage conditions [221]. These approaches seek to extend the shelf life of peptides without compromising their safety or therapeutic efficacy.

Granuloma Penetration: Another key technical challenge is the limited penetration of nanoparticles into tuberculous granulomas, whose dense, fibrous structure constitutes a difficult physical barrier to overcome. The heterogeneity of these microenvironments, characterized by necrotic regions and hypoxic zones, makes it difficult for delivery systems to reach the interior of lesions where MTB persists. To address this limitation, strategies such as particle size optimization, surface modifications that improve permeability, and combination with agents that promote disruption of the granulomatous matrix have been evaluated [230]. These advances could enhance intragranulomatous distribution and, consequently, increase treatment efficacy.

Manufacturing Scalability: Scalability in the production of peptide- and nanocarrier-based systems represents a critical challenge in their transition to clinical applications [237]. The development of industrial processes capable of ensuring consistency, reproducibility, and economic viability is complex, given that these formulations often involve specialized techniques and expensive materials. The implementation of more standardized manufacturing methods, along with automation and reductions in batch-to-batch variability, is presented as an essential condition to facilitate their regulatory approval and subsequent large-scale commercialization.

#### 7.4.2. Biological Challenges

Immunogenicity: From a biological perspective, both peptides and nanocarriers have an intrinsic potential to generate unwanted immune responses that can limit their efficacy or trigger adverse effects. Immunogenicity can arise from the nature of the peptides themselves or from the physicochemical characteristics of the materials used in the nanoparticles. Strategies to mitigate this problem include PEGylation, which reduces immune detection, the use of surface modifications that decrease immune cell activation, and the careful selection of biocompatible and non-immunogenic materials. These approaches seek to balance safety and efficacy while maintaining the therapeutic activity of the systems [222,223].

Drug Resistance: Although AMPs are less likely to generate resistance compared to conventional antibiotics, the possibility that MTB may develop adaptive mechanisms against these compounds cannot be ruled out. In particular, the selective pressure exerted by prolonged treatments could favor mutations in key genes, the overexpression of efflux pumps, or the modification of bacterial membrane components that reduce the efficacy of the peptides. Therefore, it is necessary to integrate AMPs into combined therapeutic regimens that reduce the risk of resistance and reinforce their long-term effectiveness [224].

### 7.5. Regulatory and Economic Challenges

Regulatory Pathway Complexity: The combination of innovative peptides with nanotechnology platforms creates a particularly complex regulatory landscape. Each component of the formulation requires exhaustive safety, efficacy, and quality analysis, which makes the evaluation process lengthy and expensive. Furthermore, the lack of fully standardized regulatory guidelines for such hybrid therapies adds an additional layer of difficulty. Generating robust data on toxicity, pharmacokinetics, biodistribution, and efficacy will be essential to meet the demands of regulatory agencies and advance toward clinical approval [213].

Cost Considerations: The high costs associated with both the development and production of peptide–nanocarrier systems represent a major limitation to their global application, especially in resource-limited regions where the TB burden is highest [230,237]. The use of high-value materials and specialized production processes can restrict scalability and hinder equitable access to these therapies. Therefore, it is essential to design more cost-effective manufacturing strategies and explore international collaboration models that can reduce costs and ensure the availability of these technologies in the settings where they are most needed.

Future research should focus on the design of advanced delivery systems that go beyond conventional peptide delivery. In this sense, multifunctional nanocarriers, capable of combining diagnostic, therapeutic, and monitoring properties, represent a particularly promising horizon, as they would enable not only treating the infection but also evaluating the therapy’s efficacy in real time. Similarly, the incorporation of personalized medicine approaches could allow the creation of formulations tailored to specific factors of each patient, such as their immunological profile or the particular resistance of the MTB strain they carry [230]. Another crucial aspect is the optimization of combination therapies, where AMPs are integrated with conventional antibiotics within nanocarrier systems, which would facilitate a synergistic action and reduce the likelihood of resistance [218,221].

The development of novel targeting strategies is another area of great potential. These include nanoparticles capable of effectively penetrating granulomas, either through ultra-small designs or systems that can change shape to improve their distribution in the dense, fibrous tissues characteristic of TB [230]. Furthermore, a future is envisioned in which host-directed therapy is directly integrated with the administration of AMPs, combining them with immunomodulatory agents that enhance the ability of macrophages and other immune cells to eliminate the bacteria [223,228]. Finally, the design of nanocarriers sensitive to specific TB biomarkers or bacterial metabolites opens the possibility of highly selective therapies, capable of releasing the drug only in the presence of signals associated with active infection [223].

The future of peptide–nanocarrier systems will also be shaped by the integration of emerging technologies. Artificial intelligence, and machine learning in particular, offers powerful tools for rational peptide design and formulation optimization, accelerating the discovery process and reducing reliance on empirical methods [223]. Similarly, the development of advanced characterization techniques that allow real-time monitoring of nanocarrier behavior in biological systems is emerging as a key factor in understanding their biodistribution and efficacy. Finally, theranostic systems, which combine therapeutic and diagnostic capabilities in a single platform, could transform TB management by offering personalized treatments with simultaneous response monitoring [237].

## 8. Conclusions

The global rise of MDR and XDR MTB strains underscores the urgent need for alternative therapeutic strategies. Current regimens, which rely on prolonged use of first-line antibiotics, are limited by poor patient adherence, severe side effects, and the steady emergence of resistance. In this context, host-directed therapies, particularly AMPs, represent a promising complement or substitute to conventional drugs. AMPs exhibit multiple mechanisms of action, including direct antimycobacterial activity, modulation of host immune responses, and enhancement of macrophage function. Preclinical studies demonstrate that AMPs can potentiate the efficacy of established anti-TB drugs, thereby lowering effective doses, reducing toxicity, and delaying resistance. Nonetheless, significant obstacles remain before their clinical translation. Proteolytic instability and short half-life in vivo continue to be major concerns, while issues of selectivity and potential off-target toxicity must be carefully addressed. To overcome these limitations, innovative delivery platforms are being developed, with nanotechnology offering particular promise for this purpose. Nanocarriers and inhalable systems can protect peptides from degradation, enable macrophage-specific targeting, and improve local bioavailability within the lungs—the primary site of TB infection. Future research must integrate rational peptide design, advanced delivery technologies, and rigorous clinical evaluation to fully harness the therapeutic potential of AMPs. If these challenges are addressed, peptide-based strategies may emerge as a transformative addition to the global TB treatment landscape.

## Figures and Tables

**Table 2:** Impact of Various Design Strategies on Antimicrobial Peptide Properties.

Design Strategy	Description	Impact on Peptide Properties	References
Sequence modification/alteration	Modifying amino acid sequences to optimize properties such as hydrophobicity, charge, and peptide length	Improves membrane affinity, stability, and antimicrobial activity, and reduces toxicity	[146]
Conformational Restrictions	Introducing structural modifications (e.g., cyclization, introduction of D/L-amino acids)	Enhances stability against proteolytic degradation, prolongs peptide lifespan	[98]
Lipidation	Attaching lipid groups to peptides to enhance membrane interaction	Increases membrane penetration and stability, improves antimicrobial action	[147]
Electrostatic Optimization	Modifying the charge distribution on peptides to enhance electrostatic attraction to microbial membranes	Enhances binding affinity to bacterial membranes, reduces host cell toxicity	[148]
Structural Folding	Optimizing secondary structure (e.g., alpha-helices, beta-sheets) for better functionality	Promotes proper folding, improves specificity, and reduces non-specific interactions	[149,150]
Cationic Residue Enhancement	Increasing the presence of cationic residues (e.g., lysine, arginine) to improve membrane penetration	Increases interaction with bacterial membranes, improving antimicrobial activity	[151,152]
Hydrophobicity Tuning	Modifying the hydrophobic and hydrophilic balance in peptides for better membrane interaction.	Enhances cell membrane disruption and reduces toxicity to mammalian cells.	[98,153,154]

**Table 3:** The advances in drug administration for tuberculosis treatment.

Platform/Formulation	Drugs or Molecules	Main Findings	Ref.
PLG/PLGA nanoparticles functionalized with lectins (WGA-coated PLG-NPs)	Rifampicin, Isoniazid, Pyrazinamide	More biodisponibilidad relativa; permanencia plasmática prolongada (RIF: 6–7 días; INH/PZA: 13–14 días); encapsulación 54–66%; unión de WGA ≈ 3–3.5 mg/mg NP.	[214]
Sustained-release PLG microparticles	Isoniazid + Rifampicin	Sustained release (INH: 7 weeks; RIF: 6 weeks); enhanced bacterial clearance in lungs and liver with single dose vs. daily free drug administration.	[215]
Inhalable formulations (liposomes, micro- and nanoparticles)	Several	Viable platforms for respiratory delivery; alveolar targeting; less systemic toxicity; suitable as dry powder or nebulizable formulations.	[216]
Liposomes and lipid-based particles	Rifampicin, Isoniazid	More intracellular efficacy against mycobacteria; tested with multiple antibiotics.	[217]
Oral nanoparticle formulations	NR	More stability and oral delivery; less dosing frequency in murine models.	[218]
Lipo-conjugates in polymeric NPs	Lipopeptide conjugates	Isoniazid In vitro intracellular activity; in vivo efficacy (guinea pig model); lipid conjugation + encapsulation enhance drug delivery.	[219]
Poly(ε-caprolactone) (PCL) nanoparticles	AMPs and Rifampicin	More AMP stability; synergistic activity with rifampicin in vitro.	[213]

**Table 4:** Main strategies used in the development of AMP-based nanocarriers.

Nanocarrier Type	Examples	Key Features/Advantages	Refs.
Lipid-Based Nanocomposites	Liposomes, SLNs, lipid nanocapsules	High biocompatibility; pulmonary delivery potential; efficient encapsulation of hydrophilic and lipophilic peptides; stability under aerosolization.	[217,219]
Polymeric Nanoparticles	PLGA, peptide-PEG conjugates	Sustained release; tunable degradation; surface functionalization for macrophage uptake; pH-responsive release in phagolysosomes.	[213,222,223]
Inorganic and Metal-Based Carriers	Gold NPs, silica carriers, metal–peptide complexes (Ag, Cu)	Enable surface tethering; modify peptide activity/solubility; antimicrobial synergy with silver and copper conjugates.	[221,224]
Carbon-Based Nanomaterials	Graphene quantum dots (GQDs), functionalized CNTs	Enhance intracellular antibiotic efficacy; unique penetration and trafficking properties in infected macrophages.	[218]
Self-Assembled Peptide Nanostructures	Peptide nanofibers, hydrogels	Intrinsic antimicrobial activity; controlled presentation of active sequences; biodegradable; low immunogenicity.	[223,225]

## Data Availability

Not applicable.
